# Subcutaneous administration of levothyroxine: a novel approach to refractory hypothyroidism – A review and a case report

**DOI:** 10.20945/2359-3997000000349

**Published:** 2021-04-12

**Authors:** Albert Topf, Thomas Pleininger, Lukas J. Motloch, Moritz Mirna, Kristen Kopp, Michael Lichtenauer, Uta C. Hoppe, Hermann Salmhofer

**Affiliations:** 1 Paracelsus Medical University of Salzburg Department of Internal Medicine II Salzburg Austria Department of Internal Medicine II, Paracelsus Medical University of Salzburg, Salzburg, Austria.; 2 Paracelsus Medical University of Salzburg Department of Internal Medicine I Salzburg Austria Department of Internal Medicine I, Paracelsus Medical University of Salzburg, Salzburg, Austria.

## Abstract

Treatment-refractory hypothyroidism is a common clinical finding. Substantial causes include poor compliance and intake failure as well as gastrointestinal diseases, such as inflammatory bowel disease and short bowel syndromes. Increasing oral dosage of levothyroxine (LT4) is not always effective. Therefore, alternative routes of administration are necessary. In this report, we evaluate alternative treatment modalities for refractory hypothyroidism and present a 28-year-old woman with intestinal drug malabsorption successfully treated by subcutaneous LT4 administration. In this patient, a parenteral form of LT4, 500 μg/5 ml, was administered subcutaneously in a split dosage regimen. Blood hormone levels returned to normal within a few days and remained stable over an 8-month follow-up period.

## INTRODUCTION

Up to 15-20% of patients under LT4 medication experience treatment-refractory hypothyroidism according to the literature (1). Poor adherence is the most common reason of insufficient treatment. On the other hand, malabsorption of LT4 may be caused by gastrointestinal diseases as well as interaction with concomitant medications or nutrients (2). After exclusion of poor adherence or drug interactions, an increase in dose is the standard approach for treatment. However, in some rare occasions, this approach is futile (3). In the following, we describe alternative routes of LT4 administration and present the case of a 28-year old woman with drug malabsorption successfully treated with subcutaneous injection of LT4.

## CASE REPORT

A 28-year old woman was hospitalized due to manifest hypothyroidism. Thyroid stimulating hormone (TSH) level was 41 mIU/L (range: 0.4 to 4.0 mIU/L; Electrochemiluminescence Assay, Roche, Basel, Switzerland) despite a plausible intake of 300 μg LT4 dispensed as 60 drops of aqueous LT4-sodium, 100 μg/mL in a combination with apple vinegar (L-Thyroxin Henning^®^, Sanofi-Aventis GmbH, 65926 Frankfurt/Germany; drug approval number 49804.00.00, September 26, 2003). Clinical symptoms manifested with a weight gain of 3 kilograms (body weight 68 kg, BMI 24,4), fatigue and doughy skin.

Hashimoto thyroiditis had been known since 2009 (antibody levels: thyroid peroxidase (TPO) 394 IU/mL (range: <9.0 IU/mL; Chemiluminescent Microparticle Immunoassay, Abbott, Chicago, IL, USA), thyroglobulin antibodies (TAK) 9,68 IU/mL (range: <20 IU/mL; Chemiluminescent Microparticle Immunoassay, Abbott, Chicago, IL, USA) and an autoimmune gastritis had been diagnosed by gastrointestinal biopsy in 2017. Accordingly, parietal cell antibodies were found to be positive.

All other etiologies of malabsorption, including celiac disease, exocrine pancreatic insufficiency, iron storage disease and liver failure were excluded by thorough endoscopic and laboratory investigations. There was no evidence for malnutrition or intake failure resulting from concomitant medication.

Several oral regimens of hormone replacement remained ineffective. Of note, 700 μg LT4 per day in tablet form was administered orally under observation. However, no improvement of blood hormone levels could be achieved. Furthermore, the addition of apple vinegar to an aqueous oral formulation of LT4 (L-Thyroxin Henning, see above) for suspected anacidity in autoimmune gastritis failed to improve absorption. Lack of drug adherence seemed improbable, since, a high dose of LT4 administered orally under observation in the hospital setting had not improved hormone levels, as mentioned before. On the other hand, an intravenous dose of 500 μg LT4 (L-Thyroxin Henning^®^ inject, 100 μg/mL Sanofi Aventis Gmbh, Frankfurt, Germany), rapidly normalized thyroid hormone blood levels.

Due to suspected gastrointestinal malabsorption, an alternative route of administration was needed. Subcutaneous administration of LT4 had failed in a previous report of a 42-year-old woman when performed as a single shot regimen (4). This had been due to local pain reactions at the injection site. Yet, splitting the dosage to two sites in once-weekly administration had been effective, as demonstrated by Groener and cols. (5).

**Figure 1:**
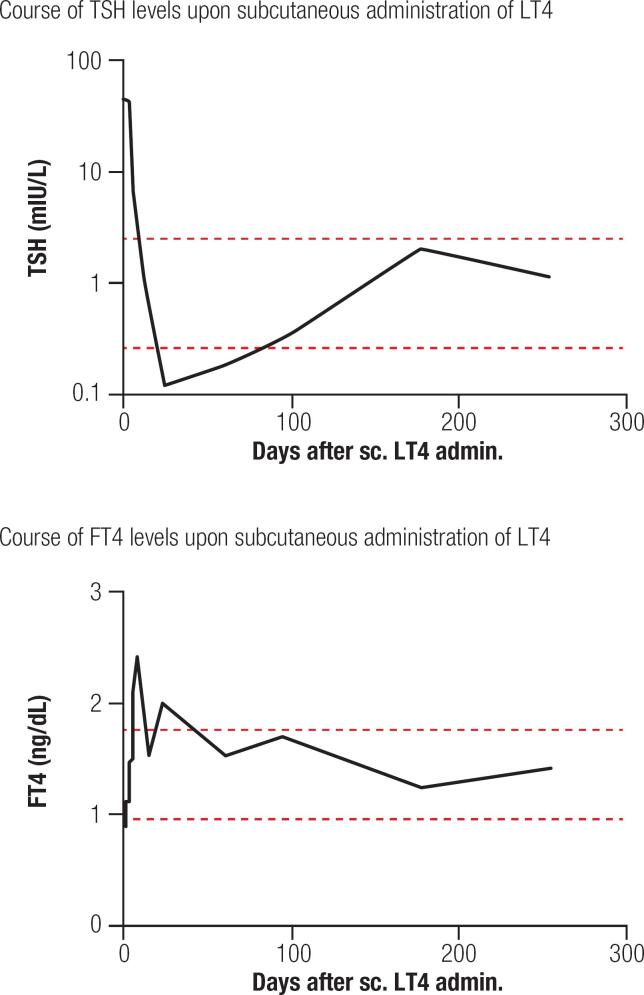
Course of TSH and FT4 levels upon subcutaneous administration of levothyroxine. Consider logarithmic scaling of TSH levels. Normal range of TSH 0.4-4 mU/L. Normal range of FT4 0,93-1,70 ng/dL.

Consequently, we started a weekly split dosage of undiluted L-Thyroxin Henning^®^ inject (500 μg/5 mL LT4, Sanofi Aventis Gmbh, Frankfurt, Germany; drug approval number 48613.00.00, September 26, 2003). Specifically, 2.5 mL volumes of L-Thyroxin Henning^®^ inject were administered subcutaneously at two separate sites of the abdomen. This side-stepped local pain reactions. After one week of therapy, an euthyroid state was achieved and maintained throughout an 8-month follow-up period (Figure 1).

In conclusion, weekly subcutaneous administration resulted in a daily LT4 demand of 71,4 μg to obtain normal thyroid hormone blood levels in this patient (resulting in a dose of 1,05 μg/kg body weight in this patient).

## DISCUSSION AND REVIEW

Hypothyroidism is usually treated by oral administration of synthetic LT4 with an average dosage of 1.4-1.6 μg/kg per day, dependent on sex and gender. Fasting intake with a glass of water thirty minutes before breakfast is generally recommended, avoiding concomitant medication at this time point. Alternatively, in some refractory patients the intake of LT4 at bedtime was shown to increase absorption (5).

Bioavailability of synthetic LT4 is 80% after oral ingestion (6). The main part of absorption takes place in the small intestine (jejunum and ileum) and is enhanced in the fasting state (7). At high age, absorption is known to be reduced (8). LT4 in blood is a pro-hormone largely bound by thyroxine-binding globulin, transthyretin and albumin (9). Subsequently, the biologically active hormone, triiodothyronine (T3), is produced by iodothyronine deiodinase in peripheral tissues on demand (10).

**Figure 2:**
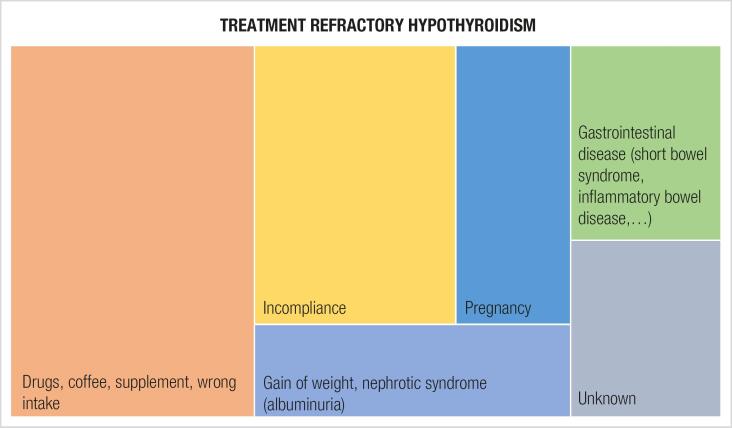
Causes of treatment refractory hypothyroidism. The prevalence is shown by a tree map. Abbreviations: Levothyroxin (LT4), subcutaneous (s.c.).

Refractory hypothyroidism is a common clinical finding. The most frequent causes are (i) poor compliance or (ii) wrong intake of LT4. Furthermore, (iii) interactions with drugs and nutritional supplements can cause hypothyroidism by malabsorption. Proton pump inhibitors, histamine receptor blockers, vitamin D and motility modifying agents as well as coffee may affect the absorption of LT4 (2). (iv) Pregnant women have an increased demand of LT4, which has its peak in mid-pregnancy. Hypothyroid women should therefore increase LT4 doses by approximately 30% once pregnancy is confirmed. The temporary chorionic gonadotropin-induced increase of the thyroxine production rate in normal gestation can provide this increment in patients with an intact thyroid function. Gestational increase in thyroxin-binding globulin is one reason for augmented requirement of LT4 in pregnancy (11) Inactivation of triiodothyronine and thyroxine by type 3 iodothyronine deiodinase, which is found widespread in the human fetoplacental unit and uterus, increases throughout pregnancy and therefore causes further requirement of LT4. Especially patients with positive anti-TPO antibody testing had higher requirement of LT4 during pregnancy when compared to anti TPO negative patients. Therefore, in women of reproductive age, pregnancy should be ruled out, if refractory hypothyroidism is manifest (10). (v) Another important factor for increased demand of LT4 is a gain of weight. Although the total daily dose of LT4 usually is higher in obese individuals, the dose per kilogram tends to be lower (2). (vi) Gastrointestinal malabsorption of LT4, as demonstrated in our case, can be caused by various diseases, such as inflammatory bowel disease, helicobacter pylori infection, celiac disease, chronic atrophic gastritis and others (7). A diagnosis of pseudo-malabsorption can only be made after exclusion of all other causes of malabsorption and is often concomitant with psychotic disorders. Variable levels of T4 and TSH, including periods of completely normal levels, would raise suspicion of pseudo-malabsorption (12). To diagnose a pseudo-malabsorption, a 1000 μg LT4 challenge test should demonstrate an increase in T4 levels two to threefold and a decrease in TSH by 40% of the initial value after two hours (3). (vii) Cystic fibrosis and a genetic variation of deiodinase are rare causes of refractory hypothyroidism. In 10-20% of patients, no reason can be detected (13,14) (Figure 2).

According to most recent guidelines, in successful treatment, TSH, as the most reliable marker for monitoring of thyroid hormone replacement, should decrease to levels of 0.4-4 mIU/L in association with a normal T4 value. Considering the different reasons for refractory hypothyroidism, several alternative routes of LT4 administration have been investigated (2) (Table 1).

**Table 1:** Overview of alternative routes of administration of levothyroxine

Route of administration	Reason for alternative administration	Dosage	Side effects	Authors	Evidence based on
Once weekly T4 oral replacement	Non compliance	Slightly larger dose than 7 times the daily dose	–	Rangan *et al.*^17^	Study including 100 people
Once weekly T4 oral replacement	Randomized crossover study	Slightly larger dose than 7 times the daily dose	–	Rajput *et al.*^19^	Study including 100 people
Once weekly T4 oral replacement	Randomized crossover study	Slightly larger dose than 7 times the daily dose	–	Grebe *et al.*^15^	Study including 12 people
Once weekly T4 oral replacement	Randomized crossover study	Slightly larger dose than 7 times the daily dose	–	Bornschein *et al.*^16^	Study including 14 people
Rectal administration (mostly suppositories)	Crisis areas, problems in health supply	Twice the oral dose	–	Kashiwagura *et al.*^21^	Study including 6 people
Rectal in the form of enema	Gastric outlet obstruction	2000 μg as enema	–	Khaled *et al.*^20^	Case report
Intramuscular	Non-compliance, gastrointestinal diseases	Twice weekly application of T4	–	Taylor^25^	Case report
Subcutaneous	Gastrointestinal diseases, possible under anticoagulation and coagulopathies	500 μg in a split dose	–	Groener *et al.*^5^	Case report
Subcutaneous	Malabsorption	500 μg in one application site	Painful local reaction due to application of entire dose to one place	Nobre *et al.*^4^	Case report
Weekly intravenous LT4	Non compliance, gastrointestinal disease	300 μg of LT4 diluted in 50 mL of saline, weekly by intravenous infusion over 15 minutes	–	Nakano^22^	Case report

Due to the relatively long half-life of T4 (5-7 days), once weekly LT4 oral replacement is a safe, effective and well-tolerated treatment for patients with non-compliance. For complete biochemical normalisation, a slightly larger dose than 7 times the daily dose may be required in weekly oral intake. T3 and T4 levels are elevated for 2 hours after intake and then remain within normal ranges for 8 days. At the peripheral tissue level, the effects of weekly treatment do not differ from customary daily treatment. No obvious adverse effects have been reported so far, yet large clinical trials including elderly patients and those with a history of ischemic heart disease are lacking (15-19).

Rectal administration of LT4 has been tried mostly in the form of suppositories. Experiences from animal models and previous human reports demonstrated that high doses, ranging between 31.25 μg/kg and 62.5 μg/kg, were required for an adequate response. LT4 suppositories showed lower bioavailability and efficacy when compared to oral LT4. Yet, sufficient T4 levels could be maintained in patients with hypothyroidism by administering LT4 suppositories at approximately twice the oral dose. The lower bioavailability of rectal LT4 compared to its oral counterpart has been attributed to the low level of LT4 release in the rectum. In reports from developing countries, when injectable LT4 was unavailable, even rectal enemas have been administered successfully (20,21).

Intravenous LT4 administration has been used in medical emergencies such as myxedema. Its efficacy in refractory hypothyroidism has only been reported in single cases so far. In a previous report, T4 sodium salt pentahydrate had been dissolved by 0.1 N NaOH solution and diluted to a concentration of 200 μg/2 mL by saline. A total of 300 μg of LT4 diluted in 50 mL of saline had been administered once weekly by intravenous infusion over 15 minutes. Pharmacokinetics of once-weekly intravenous administration were similar to those of daily oral administration and thyroid hormone levels could be maintained in the normal range without adverse events (22-24).

Intramuscular administration of LT4 was shown to be a successful, alternative route in other reports. With a once weekly dose, a variability of T4 levels had been observed, which could be reduced by using a twice-weekly regimen (25).

To the best of our knowledge, two reports of subcutaneous LT4 administration have been published (4,5). The first case failed because of painful local reaction due to injection of the entire dose of 10 mL at one single site. In a second report of 2013, a split dosage using two injection sites was successful. The major advantage of subcutaneous application is simple handling, which can be done by the patients themselves. Furthermore, it can be performed in patients under anticoagulation and with coagulopathies. Similar to the preceding report, we demonstrated that off-label use of subcutaneous LT4 is safe and effective in patients with refractory hypothyroidism. Subcutaneous injection of drugs, as in other diseases like diabetes, seems to be the most suitable route of administration in persons with malabsorption of LT4. This may be particularly important for persons with inflammatory bowel disease, short bowel syndrome and total parenteral nutrition (4).

## CONCLUSION

Among alternative approaches of thyroid hormone replacement, subcutaneous administration proved to be simple, safe and effective. Therefore, we suggest that this approach, using the split dosage regimen, should be preferred in cases of refractory hypothyroidism.

## References

[1] Benvenga S, Papi G, Antonelli A (2017). Refractory Hypothyroidism Due to Improper Storage of Levothyroxine Tablets. Front Endocrinol (Lausanne).

[2] Centanni M, Benvenga S, Sachmechi I (2017). Diagnosis and management of treatment-refractory hypothyroidism: an expert consensus paper. J Endocrinol Invest.

[3] Van Wilder N, Bravenboer B, Herremans S, Vanderbruggen N, Velkeniers B (2017). Pseudomalabsorption of levothyroxine: A challenge for the endocrinologist in the treatment of hypothyroidism. Eur Thyroid J.

[4] Nobre EL, Jorge Z, Anselmo J, Lopes C, César R, Santos V (2004). A rare case of malabsorption of thyroid hormones. Acta Med Port.

[5] Groener JB, Lehnhoff D, Piel D, Nawroth PP, Schanz J, Rudofsky G (2013). Subcutaneous application of levothyroxine as succesful treatment option in a patient with malabsorption. Am J Case Rep.

[6] Balla M, Jhingan RM, Rubin DJ (2015). Rapid Levothyroxine Absorption Testing: A Case Series of Nonadherent Patients. Int J Endocrinol Metab.

[7] Virili C, Antonelli A, Santaguida MG, Benvenga S, Centanni M (2019). Gastrointestinal Malabsorption of Thyroxine. Endocrine Reviews.

[8] Calsolaro V, Niccolai F, Pasqualetti G, Tognini S, Magno S, Riccioni T (2019). Hypothyroidism in the Elderly: Who Should Be Treated and How?. J Endocr Soc.

[9] Schussler GC (2000). The thyroxine-binding proteins. Thyroid.

[10] St Germain DL, Galton VA, Hernandez A (2009). Minireview: Defining the roles of the iodothyronine deiodinases: current concepts and challenges. Endocrinology.

[11] Alexander EK, Marqusee E, Lawrence J, Jarolim P, Fischer GA, Larsen PR (2004). Timing and magnitude of increases in levothyroxine requirements during pregnancy in women with hypothyroidism. N Engl J Med.

[12] Livadariu E, Valdes-Socin H, Burlacu MC, Vulpoi C, Daly AF, Beckers A (2007). Pseudomalabsorption of thyroid hormones: case report and review of the literature. Ann Endocrinol (Paris).

[13] Chesdachai L, Braverman T (2016). Thyroid Function in Patients with Cystic Fibrosis: No Longer a Concern?. Thyroid.

[14] Gereben B, McAninch EA, Ribeiro MO, Bianco AC (2015). Scope and limitations of iodothyronine deiodinases in hypothyroidism. Nat Rev Endocrinol.

[15] Grebe SKG, Cooke RR, Ford HC, Fagerström, JN, Cordwell DP, Lever NA (1997). Treatment of hypothyroidism with once weekly thyroxine. J Clin Endocrinol Metab.

[16] Bornschein A, Paz G, Graf H, Carvalho GA (2012). Treating primary hypothyroidism with weekly doses of levothyroxine: a randomized, single-blind, crossover study. Arq Bras Endocrinol Metabol.

[17] Rangan S, Tahran AA, Macleod AF, Moulik PK (2007). Once weekly thyroxine treatment as a strategy to treat non-compliance. Postgrad Med J.

[18] Doraiswamy A, Zainudin S (2014). Efficacy and cardiac safety of short term weekly levothyroxine administration in hypothyroid patient on replacement therapy.

[19] Rajput R, Pathak V (2017). The Effect of Daily versus Weekly Levothyroxine Replacement on Thyroid Function Test in Hypothyroid Patients at a Tertiary Care Centre in Haryana. Eur Thyroid J.

[20] Khaled A, Obeidat NA, Saadeh A, Sohail B (2018). Successful Management of Hypothyroidism in Gastric Outlet Obstruction Using Levothyroxine Rectal Enemas: A Case Report. Am J Case Rep.

[21] Kashiwagura Y, Uchida S, Tanaka S, Watanabe H, Masuzawa M, Sasaki T (2014). Clinical efficacy and pharmacokinetics of levothyroxine suppository in patients with hypothyroidism. Biol Pharm Bull.

[22] Nakano Y (2019). A case of refractory hypothyroidism due to poor compliance treated with weekly intravenous and oral levothyroxine administration. Case Reports in Endocrinology.

[23] Hays MT (2007). Parenteral Thyroxine Administration. Thyroid.

[24] Miyauchi A, Kataoka K, Suzuki Y, Kishi H, Takai S, Okagawa K, Maeda M, Kosaki G (1984). Parenteral replacement of thyroid hormones. Nihon Naibunpi Gakkai Zasshi.

[25] Taylor PN, Tabasum A, Sanki G, Burberry D, Tennant BP, White J (2015). Weekly Intramuscular Injection of Levothyroxine following Myxoedema: A Practical Solution to an Old Crisis. Case Rep Endocrinol.

